# Phylogeny of *Aspergillus* section *Circumdati* and inhibition of ochratoxins potential by green synthesised ZnO nanoparticles

**DOI:** 10.1080/21501203.2024.2379480

**Published:** 2024-08-27

**Authors:** Mohamed A. Hussein, Youssuf A. Gherbawy, Mahmoud S. Abd El-sadek, Helal F. Al-Harthi, Eman GAM El-Dawy

**Affiliations:** a Botany and Microbiology Department, Faculty of Science, South Valley University, Qena, Egypt; b Applied and Environmental Microbiology Center, South Valley University, Qena, Egypt; c Physics Department, Faculty of Science, South Valley University, Qena, Egypt; d Department of Physics, Faculty of Science, Galala University, Suez, Egypt; e Biology Department, Turabah University College, Taif University, Taif, Saudi Arabia

**Keywords:** Phylogenetic, *Aspergillus*, *Circumdati*, *pks* gene, ZnO-NPs, *Ocimum basilicum*

## Abstract

Contamination of agricultural and industrial products by *Aspergillus* section *Circumdati* becomes a true problem, especially, because a lot of species in this section can yield ochratoxins and other toxins. In this study, morphological criteria and partial calmodulin gene were used to identify 34 strains belonging to *Aspergillus* section *Circumdati* isolated from *Vitis vinifera* and *Calotropis procera* plants. The population is characterised by *A*. *insulicola*, *A*. *ochraceopetaliformis*, *A*. *ochraceus*, and *A*. *pseudoelegans*. The polyketide synthase (*pks*) gene involved in ochratoxins (OTA) production was investigated by *Aopks*1, 2 and *AoLc*35-12L, R primers. Fifteen strains belonging to *A*. *ochraceus* were positive for *pks* gene, whilst *A*. *insulicola*, *A*. *ochraceopetaliformis*, and *A*. *pseudoelegans* were negative. All tested strains were able to produce ochratoxin A with different levels of 0.020–53 ppm except one isolate of *A*. *ochraceus* (IAEMAo6). The green synthesised ZnO-NPs have a significant inhibitory effect on OTA production by *A. insulicola* and *A*. *pseudoelegans*.

## 1. Introduction

*Aspergillus* is considered the most abundant fungi worldwide; it comprises 20 different sections (Houbraken et al. 2014; Hubka et al. 2015). From which *Aspergillus ochraceus* group or *A*. section *Circumdati* comprises fungal species in yellow to ochre shades with biseriate conidial heads (Visagie et al. 2014). This section contaminates several agricultural and industrial products (Pandit et al. 2015; El-Hamaky et al. 2016). The early study of section *Circumdati* was conducted by Raper and Fennell (1965), they revealed that this section included nine species (*A*. *alliaceus*, *A*. *auricomus*, *A*. *elegans*, *A*. *fresenii*, *A*. *melleus*, *A*. *ochraceus*, *A*. *ostianus*, *A*. *petrakii*, and *A*. *sclerotiorum*), after that numerous new species were described as members of this section (Christensen and Raper 1978; Christensen 1982; Zotti and Corte 2002). Based on morphological data and partial *β*-tubulin, seven new species (*A*. *cretensis*, *A*. *flocculosus*, *A*. *neobridgeri*, *A*. *pseudoelegans*, *A*. *roseoglobulosus*, *A*. *steynii*, and *A*. *westerdijkiae*) were added to the section (Frisvad et al. 2004). Visagie et al. (2014) revised section *Circumdati*; they reported that 27 species were accepted in this section and *A*. *flocculosus* was considered synonymous with *A*. *ochraceopetaliformis*, whilst *A*. *onikii* and *A*. *petrakii* belonged to *A*. *ochraceus*. Recently, *A. curvatus* a new species was added to section *Circumdati* (Al-Bedak et al. 2020).

Numerous species of *A*. *ochraceus* group are well known for their production of several mycotoxins including ochratoxins A (Robbers et al. 1978; Bayman et al. 2002; Dao et al. 2005). Visagie et al. (2014) concluded that *A*. *ochraceopetaliformis* (*A*. *flocculosus*), *A*. *ochraceus*, *A. pseudoelegans*, and other 10 species of section *Circumdati* produce large quantities of ochratoxins A. The OTA biosynthetic pathway is believed that formed via polyketide synthesis pathway (Moss 1998) and nonribosomal peptide synthetases (Gallo et al. 2013). Several attempts aimed to identify genes involved in OTA biosynthesis. O’Callaghan et al. (2003) cloned part of the polyketide synthase (*pks*) gene involved in OTA production by *A*. *ochraceus*. Recently, different primers were used to detect OTA biosynthesis genes in different fungal species (O’Callaghan et al. 2006; Algammal et al. 2021).

Nanoparticles have tremendous applications in different science branches. Among nanoparticles, zinc oxide (ZnO-NPs) earns special attention as a strong antimicrobial agent (Sawai 2003; He et al. 2011). Green synthesis of ZnO-NPs has lower hazards to the environment and is recognised as safe by the American Food and Drug Administration (Lopes de Romana et al. 2002).

The study aims to identify the collected *Aspergillus* sect. *Circumdati* population, and investigate the presence of *pks* gene, quantitative assessment of ochratoxins level and anti-mycotoxin effect of ZnO-NPs.

## 2. Materials and methods

### 2.1. Collected isolates

Thirty-four isolates were isolated from *Vitis vinifera* and *Calotropis procera* plants. The morphological data were obtained by culturing the tested strains on Malt Extract Agar (MEA) (Oxide) at 25 °C for 7 days. Macroscopic (colony colour, reverse, texture, and diameter) and microscopic data (stipes, vesicles, phialides, and conidia) were recorded.

### 2.2. *DNA isolation from* Aspergillus *strains*

*Aspergillus* section *Circumdati* isolates were grown on PDA medium for 2–3 days at 28 °C. The grown mycelium was harvested and A cetyl trimethyl ammonium bromide buffer (CTAB) protocol was used to extract DNA as mentioned by Moeller et al. (1992).

### 2.3. PCR amplification and DNA sequencing

A partial sequence of the Calmodulin gene (*CaM*) was done to identify *Aspergillus* isolates at the species level. The PCR reaction was carried out by primers CF1 and CF4 primers (Table 1). The reaction was done in a tube containing a total volume of 20 µL: 10 µL of 2× Taq master (Jena Bioscience), 0.5 µL from each primer, 1 µL of the DNA, and 8 µL PCR water. The amplification condition was initial denaturation at 94 °C for 5 min followed by 35 cycles of denaturation at 94 °C for 45 s, annealing at 55 °C for 45 s, and extension at 72 °C for 1 min, followed by a final extension step at 72 °C for 10 min. The PCR product was checked in 1.4% agarose gel stained with ethidium bromide, then purified and sequenced in Macrogen (South Korea).

**Table 1.** List of primers used in this study.

Gene	Primers	Sequence (5′-3′)	Reference
Calmodulin (*CaM*)	CF1 CF4	5′-GCC GAC TCT TTG ACY GAR GAR-3′ 5′-TTT YTG CAT CAT RAG YTG GAC-3′	Peterson et al. (2005)
Polyketide synthase gene (*pks*)	*Aopks*1 *Aopks*2	5′-CAG ACC ATC GAC ACTGCA TGC-3′ 5′-CTG GCG TTC CAG TAC CATGAG-3′	Reddy et al. (2013)
	*AoLc*35-12 L *AoLc*35-12 R	5′-GCCAGACCATCGACACTGCATGCTC-3′ 5′-CGACTGGCGTTCCAGTACCATGAGCC-3′	Dao et al. (2005)

### 2.4. Phylogenetic analysis

The obtained sequences of the Calmodulin gene were edited using Chromas Lite and aligned using BioEdit, a ClustalW software. The phylogenetic tree with bootstrap values was constructed using MEGA software 6.0 (Tamura et al. 2013). *Aspergillus tanneri* is closely related to *A*. section *Circumdati*, therefore tree was rooted by *A*. *tanneri* JN896583.

### 2.5. *Detection of polyketide synthase gene* (pks) *of* A. *section* Circumdati

Two sets of primers were used for the amplification of *pks* gene (Table 1). The reaction was done in a tube containing 10 μL 2× Taq master (Jena Bioscience), 0.5 μL of each primer, 1 μL template DNA, and the volume completed by deionised water to 20 μL. PCR program for *Aopks*1, 2 started by denaturing at 94 °C for 5 min, followed by 30 cycles at 94 °C for 1 min, 58 °C for 1 min, 72 °C for 1 min, and final elongation at 72 °C for 10 min (Reddy et al. 2013). In the case of *AoLc*35-12 L, R the conditions were: initial denaturation at 94 °C for 4 min, followed by 35 cycles at 94 °C for 40 s, 58 °C for 40 s, 72 °C for 40 s, and final extension at 72 °C for 10 min (Dao et al. 2005). The obtained PCR products were checked on 1.2% agarose gel in 1× TAE buffer, stained with ethidium bromide, and scanned using the Gel Documentation & Analysis system (Geldoc 3.2).

### 2.6. *Quantitative determination of ochratoxins production by* Aspergillus *strains*

The ochratoxins potential by collected *Aspergillus* was estimated according to Gabal et al. (1994). Active growing disc from 7 days culture was transferred to a conical flask containing 100 mL of yeast extract sucrose (YES) broth with constituents (g/L): sucrose 40 g and yeast extract 20 g. After 2 weeks of incubation at 28 °C, the fungal filtrates were separated by filtration and then refiltrated through glassfiber paper. The amounts of OTA were estimated in 10 mL that passed through ochratoxins column (VICAM, Watertown, MA, USA) at 1–2 drops/s. The columns were washed twice with 10 mL of deionised water and the ochratoxins were eluted using 1.5 mL ochratoxins elution solution. The concentration of ochratoxins was read on a recalibrated VICAM series-4 Fluorometer (Hussein and Gherbawy 2019).

### 2.7. Anti-mycotoxin effect of zinc oxide nanoparticles

#### 2.7.1. Biosynthesis and characterization of ZnO-NPs

Zinc oxide nanoparticles were synthesised according to Faisal et al. (2021) with some modifications. 20 mL *Ocimum basilicum* extract (6%) were added dropwise to a conical flask containing 50 mL 0.2 mol/L zinc acetate dehydrate (Sigma-Aldrich) under continuous stirring then the pH of the solution was adjusted to 11.5 by NaOH (1 mol/L), afterwards, the solution heated for 60 min at 60 °C. The mixture was allowed to ageing and centrifuged for 10 min at 1,000 r/min. The collected pellet was washed twice with distilled water, once with 70% ethanol, dried in an oven at 35 °C, and calcination was made at 500 °C for 120 min.

Characterization methods including UV spectroscopy and X-ray diffraction (XRD) were used to detect the physiochemical properties of synthesised ZnO-NPS.

#### 2.7.2. *Effect of ZnO-NPs on ochratoxins production by* A. insulicola *and* A. pseudoelegans

The efficacy of ZnO-NPs against ochratoxins production was assayed by adding 1% of ZnO-NPs to a conical flask containing 100 mL of YES medium and active growing fungal disc, and then the flask was incubated at 28 °C for 15 days. Flasks without ZnO-NPs are considered as controls. After incubation, flasks were filtered and ochratoxins amounts were estimated as previously mentioned (Hussein and Gherbawy 2019).

## 3. Results

### 3.1. *Morphological characterization of* A. *section* Circumdati *population*

**Figure 1.**
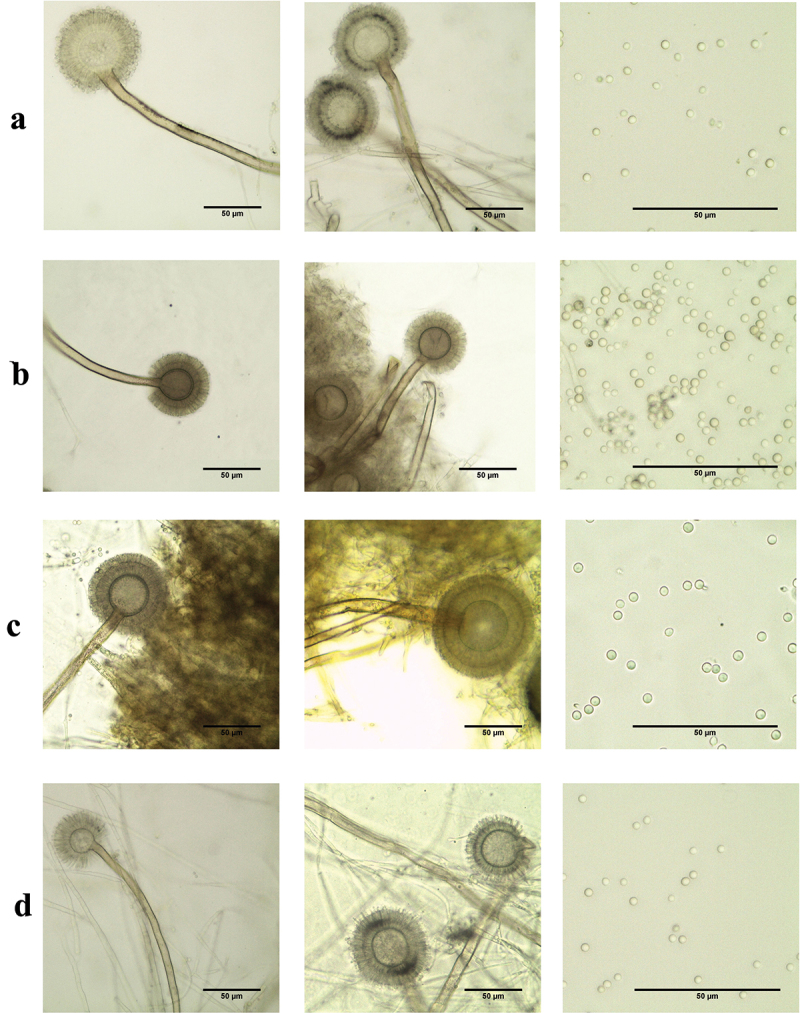
Photomicrographs showing conidiophore, vesicle, sterigmata, and conidia of *Aspergillus* section *Cicumdati* strains. (a) *A*. *insulicola*. (b) *A*. *ochraceopetaliformis*. (c) *A*. *ochraceus*. (d) *A*. *pseudoelegans*.

**Figure 2.**
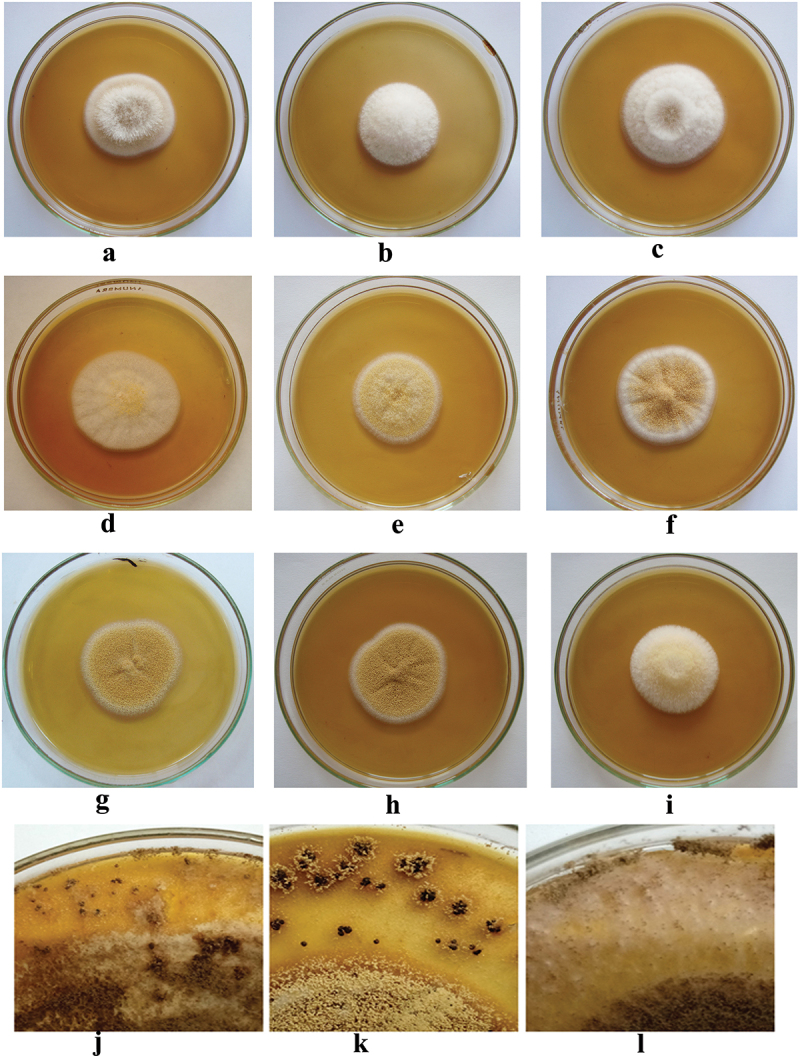
Photographs showing colony features and sclerotia of *Aspergillus* section *Circumdati* strains. (a) *A*. *insulicola*. (b, c, and j) *A*. *ochraceopetaliformis*. (d–h and k) *A*. *ochraceus*. (i and l) *A*. *pseudoelegans*.

Thirty-four strains isolated from different sources were subjected to morphological description by observing micro and macroscopical features on MEA medium (Table 2 and Figures 1, 2). Based on the obtained data, strains were characterised into the following species:

**Table 2.** Macro and microscopic criteria of *Aspergillus* section *Circumdati* on MEA medium at 25 °C for 1 week.

	*A*. *insulicola*	*A*. *ochraceopetaliformis*	*A*. *ochraceus*	*A*. *pseudoelegans*
Colony				
Color	White with greyish orange edge	White to crème grey	Light yellow to greyish yellow	Light yellow
Diameter (mm)	39–41	36.5–44.25	36.5–46.5	35–37
Texture	Floccose surface	Floccose surface	Velutinous with floccose areas in some strains	Floccose surface
Reverse	Light yellow	Light brown to brown	Light brown to dark brown	Brownish orange
Stipe				
Texture and colour	Rough, hyaline to brown	Rough, yellow to brown	Rough, hyaline to brown	Rough, hyaline to yellow
Stipe (width µm)	7.12–10.11	6.33–9.22	7.75–12.53	7.80–10.36
Vesicle				
Vesicle shape	Globose	Globose to pyriform	Globose	Globose to spathulate
Vesicle size (µm)	22.6–29.1	24.28–35.16	26.76–46.4	27.492–31.13
Conidia				
Conidail	Smooth	Smooth	Fine roughened	Smooth
Shape	Globose to subglobose	Globose	Globose to subglobose	Globose
Size	2.1–2.2 × 2.1–2.8	1.7–3.1 × 1.9–3.4	2.1–3.0 × 2.2–3.4	2.1–2.5 × 2.1–2.6
Sclerotia	Absent	Reddish brown	Purplish brown	White to greyish

#### 3.1.1. A. insulicola

The colony on MEA plate after 7 days of incubation at 25 °C was approximately 39–41 mm in diam., with floccose surface; mycelia areas white; sporulation greyish orange at the edge of colony; exudate absent; reverse light yellow; vesicles globose and biseriate, 25.9–30.0 µm size; stipes hyaline to brown, rough walled, 8.93–9.37 µm width; conidia were globose to subglobose, smooth, 2.1–2.2 × 2.1–2.8 µm; sclerotia not found.

#### 3.1.2. A. ochraceopetaliformis

The colony ranged from 36.5 to 44.25 mm in diam., their appearance typically dominated by white mycelia (IAEMAop1, 2, 4, and 5) or white to crème grey (IAEMAop3); sporulation was formed after more incubation time as olive brown; reverse light brown (IAEMAop1, 2, and 4), brown (IAEMAop3 and 5). The conidial head was biseriate; vesicles were globose-pyriform shape with 24.28–35.16 µm size; stipes yellow to brown colour, rough, 6.33–9.22 µm width; the conidia were smooth and globose, 1.7–3.1 × 1.9–3.4 µm size. Reddish-brown sclerotium-like structures were formed after prolonged incubation.

#### 3.1.3. A. ochraceus

The colony surface was velutinous with floccose areas in some strains, their sizes ranged from 36.5 to 46.5 mm; sporulation light yellow to greyish yellow; exudates present in strains (IAEMAo1, 9, 15, 21, 22, and 27) and absent in the rest of strains; reverse brown to dark brown centre (IAEMAo1 and 18), brown (IAEMAo10, 12, 15, and 21), light brown in other strains. Vesicles biseriate, globose, 26.76–46.4 µm size; stipes hyaline to brown, rough walled, 7.75–12.53 µm width; conidia globose to subglobose, finely roughened, 2.1–3.0 × 2.2–3.4 µm size; sclerotia were purplish brown.

#### 3.1.4. A. pseudoelegans

The colony was 35–37 mm in size; surface floccose with light yellow mycelia; sporulation after prolonged time; reverse brownish orange; conidiophores biseriate; vesicle globose, 27.492–31.13 µm; stipes rough walled, hyaline to yellow colour; 7.80–10.36 µm width; conidia were smooth, globose to subglobose, 2.1–2.5 × 2.1–2.6 µm size; after a long time of incubation, white to greyish sclerotia covered by mycelium were formed.

### 3.2. *Phylogenetic analysis of* A. *section* Circumdati *population*

**Figure 3.**
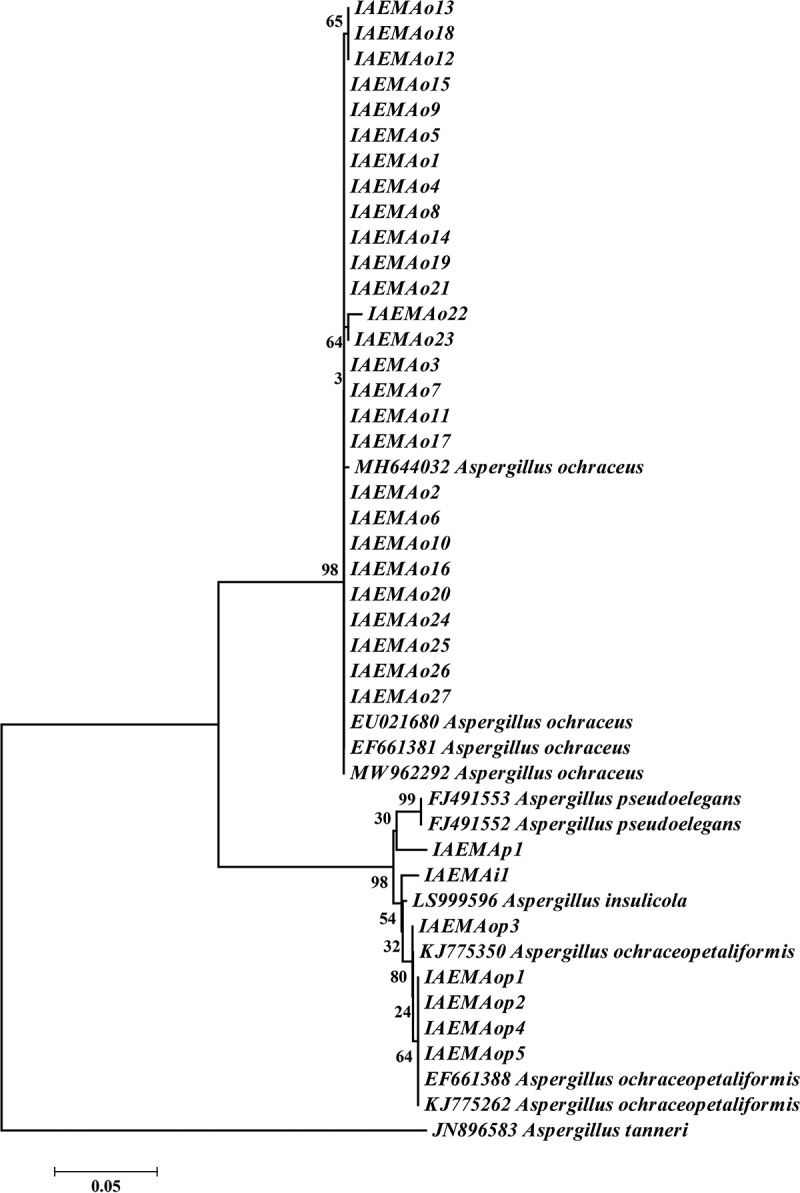
Phylogenetic tree inferred from Neighbor-Joining analysis of *Aspergillus* section *Circumdati* strains based on partial calmodulin gene, the numbers beside the nodes represent the bootstrap values out of 1,000 bootstrap replications and rooted by *Aspergillus tanneri* (JN896583).

The partial sequences of the Calmodulin (*CaM*) gene for tested strains showed 97%–100% similarity to GenBank depositing strain belonging to *A*. section *Circumdati* species. The created analysis revealed the characterisation of the collected population into 4 different species. The phylogenetic analysis showed splitting the population into two main clades. The former one consisted of 27 strains (IAEMAo1–IAEMAo27) which clustered with different *A*. *ochraceus* strains obtained from GenBank with bootstrap 98. The remaining 7 strains formed the second clade, from which five strains (IAEMAop1–5) clustered with *A*. *ochraceopetaliformis* strains (KJ775350, EF661388, and KJ775262) obtained from GenBank with bootstrap 80. IAEMAi1 and IAEMAp1 strains created separated groups with *Aspergillus insulicola* and *A*. *pseudoelegans* strains obtained from GenBank, but with relatively low bootstrap (Figure 3).

### 3.3. *Detection of polyketide synthase gene* (pks) *of* A. *section* Circumdati

**Figure 4.**
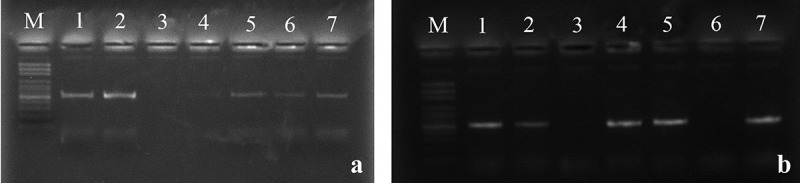
Specific detection of polyketides synthetase (*pks*) genes. (a) *Aopks*1, 2 primers, lanes; 1–7 represent the strains IAEMAo5, 6, 7, 8, 9, 10, and 11. (b) *AoLc*35-12 L, R primers, Lanes; 1–7 represent the strains IAEMAo5, 6, 7, 9, 10, 11, and 12.

The polyketide synthase gene (*pks*) was detected using two primer pairs (*Aopks*1, 2 and *AoLc*35-12 L, R). The data indicated that all positive strains belonged to *A*. *ochraceus* species, and the rest species failed to amplify with *Aopks*1, 2, and *AoLc*35-12 L, R primers. Eleven strains (IAEMAo4, 5, 6, 9, 10, 12, 14, 17, 18, 25, and 27) amplified the two tested regions in *pks* gene with 549 and 520 bp bands, respectively. Three strains IAEMAo8, 19, and 22 amplified with *AoLc*35-12 L, R primer producing 520 bp fragments but failed to give any detectable band with *Aopks*1, 2. Strain (IAEMAo11) succeeded in amplifying only *Aopks*1, 2 (Figure 4).

### 3.4. *Ochratoxins potential of* A. *section* Circumdati

Approximately 97% of *Aspergillus* strains were ochratoxin A producers with amounts ranging from 0.020 to 53 ppm. The highest level (53 ppm) was achieved by IAEMAp1 (*A*. *pseudoelegans*), followed by IAEMAi1 (*A. insulicola*) and IAEMAop2 (*A*. *ochraceopetaliformis*) with an amount of 27 ppm for each. The ochratoxins production by *A. ochraceopetaliformis* strains was 3.8–27 ppm, and the lowest level was achieved by IAEMAop4. All strains attributed to *A*. *ochraceus* were ochratoxins producers except IAEMAo6; the maximum amount of ochratoxins in this group (20 ppm) was achieved by IAEMAo24 isolated from *V. vinifera*, while the lowest one (0.02) was IAEMAo20 from *C. procera* plant (Table 3).

**Table 3.** Strains code, species name, source of isolation, accession numbers, OTA levels, *Aopks*1, 2 and *AoLc*35-12 L, R genes of *Aspergillus* section *Circumdati* strains.

Strain code	Species name	Source of isolation	Accession number	OTA (ppm)	*Aopks*1, 2	*AoLc*35-12 L, R
IAEMAi1	*A. insulicola*	*V. vinifera*	PP069281	27	-	-
IAEMAop1	*A. ochraceopetaliformis*	*V. vinifera*	PP069283	15	-	-
IAEMAop2	*A. ochraceopetaliformis*	*V. vinifera*	PP069284	27	-	-
IAEMAop3	*A. ochraceopetaliformis*	*V. vinifera*	PP069285	19	-	
IAEMAop4	*A. ochraceopetaliformis*	*V. vinifera*	PP069286	3.8	-	-
IAEMAop5	*A. ochraceopetaliformis*	*V. vinifera*	PP069287	6.6	-	-
IAEMAo1	*A. ochraceus*	*V. vinifera*	PP083981	3.4	-	-
IAEMAo2	*A. ochraceus*	*V. vinifera*	PP083982	5.7	-	-
IAEMAo3	*A. ochraceus*	*V. vinifera*	PP083983	6.6	-	-
IAEMAo4	*A. ochraceus*	*V. vinifera*	PP083984	1.1	+	+
IAEMAo5	*A. ochraceus*	*V. vinifera*	PP083985	3.4	+	+
IAEMAo6	*A. ochraceus*	*V. vinifera*	PP083986	0	+	+
IAEMAo7	*A. ochraceus*	*V. vinifera*	PP083987	0.18	-	-
IAEMAo8	*A. ochraceus*	*V. vinifera*	PP083988	9.5	-	+
IAEMAo9	*A. ochraceus*	*V. vinifera*	PP083989	0.29	+	+
IAEMAo10	*A. ochraceus*	*V. vinifera*	PP083990	3.9	+	+
IAEMAo11	*A. ochraceus*	*V. vinifera*	PP083991	3.3	+	-
IAEMAo12	*A. ochraceus*	*V. vinifera*	PP083992	15	+	+
IAEMAo13	*A. ochraceus*	*V. vinifera*	PP083993	15	-	-
IAEMAo14	*A. ochraceus*	*V. vinifera*	PP083994	7.3	+	+
IAEMAo15	*A. ochraceus*	*V. vinifera*	PP083995	1.4	-	-
IAEMAo16	*A. ochraceus*	*V. vinifera*	PP083996	0.37	-	-
IAEMAo17	*A. ochraceus*	*V. vinifera*	PP083997	19	+	+
IAEMAo18	*A. ochraceus*	*V. vinifera*	PP083998	13	+	+
IAEMAo19	*A. ochraceus*	*C. procera*	PP083999	6.6	-	+
IAEMAo20	*A. ochraceus*	*C. procera*	PP084000	0.02	-	-
IAEMAo21	*A. ochraceus*	*V. vinifera*	PP084001	2.2	-	-
IAEMAo22	*A. ochraceus*	*V. vinifera*	PP084002	5.2	-	+
IAEMAo23	*A. ochraceus*	*C. procera*	PP084003	1.4	-	-
IAEMAo24	*A. ochraceus*	*V. vinifera*	PP084004	20	-	-
IAEMAo25	*A. ochraceus*	*V. vinifera*	PP084005	3	+	+
IAEMAo26	*A. ochraceus*	*V. vinifera*	PP084006	2.9	-	-
IAEMAo27	*A. ochraceus*	*V. vinifera*	PP084007	3.9	+	+
IAEMAp1	*A. pseudoelegans*	*V. vinifera*	PP069282	53	-	-

### 3.5. Anti-mycotoxin effect of zinc oxide nanoparticles

#### 3.5.1. Characterization of ZnO-NPS

**Figure 5.**
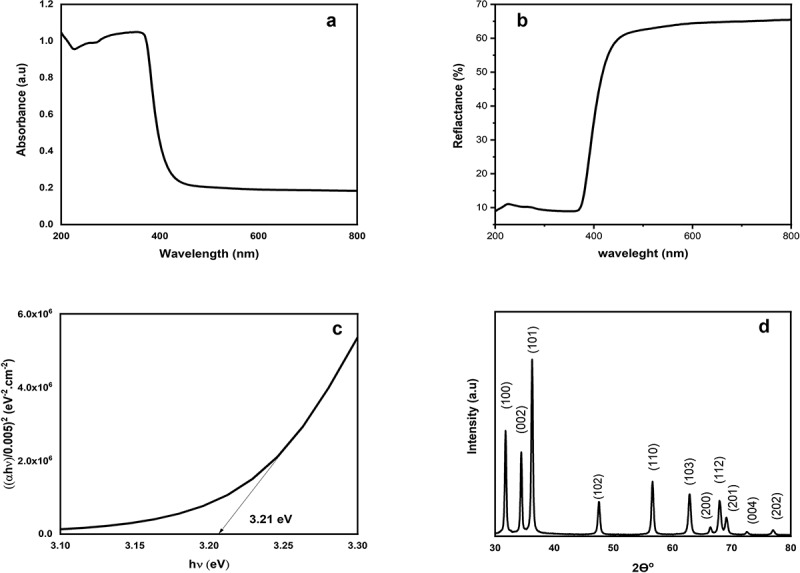
(a) UV band. (b) UV-vis diffuse reflectance spectra. (c) UV band gap. (d) XRD pattern of green synthesised ZnO-NPs.

The biosynthesis of ZnO-NPs was done using an aqueous extract of *Ocimum basilicum* as a reducing agent. Using UV-visible diffuse reflectance spectroscopy in the 200–800 nm range, the absorption peaks of ZnO-NPs were detected between 300 and 400 nm (Figure 5(a)). A high reflecting feature around 440 nm and a straight band gap of 3.21 eV was shown for ZnO-NPs sample (Figure 5(b,c)). XRD data revealed that the diffraction peaks located between 32 and 77.5 have been indexed as hexagonal wurtzite structures. The diffraction peaks were sharper and stronger due to the absence of contaminant and crystallite quality of the nanoparticles. According to Scherer’s equation (*D* = *kλ*/*β* cos *θ*), the average crystalline size of ZnO-NPs was approximately 23.1 nm (Figure 5(d)).

#### 3.5.2. *Effect of ZnO-NPs on ochratoxins production by* A. insulicola *and* A. pseudoelegans

**Figure 6.**
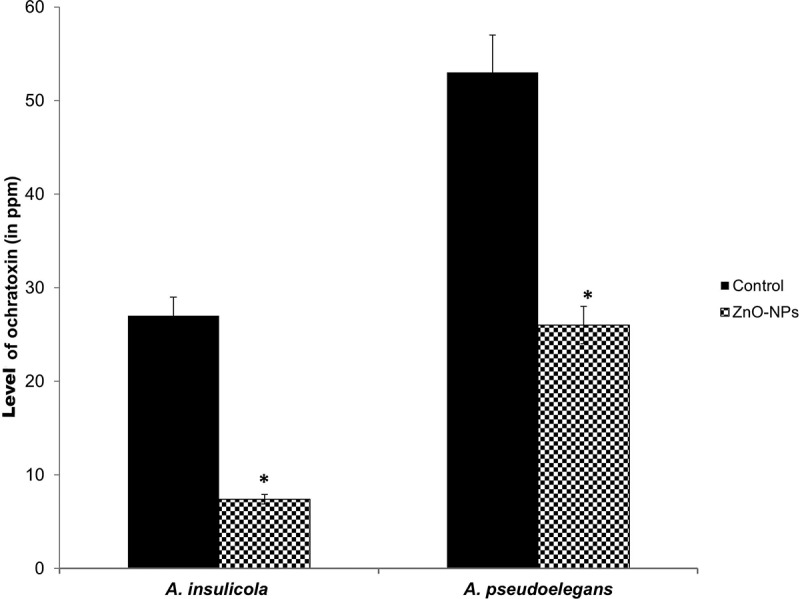
Effect of ZnO-NPs on ochratoxin production by *Aspergillus insulicola* and *A*. *pseudoelegans*. *Means significant at *p* < 0.05.

Data in Figure 6 explained that ZnO-NPs have a great inhibition effect on ochratoxin A production by tested fungi with the highest ochratoxins potentials. The inoculation of *A*. *insulicola* and *A*. *pseudoelegans* cultures with ZnO-NPs greatly reduced the toxin level from 27 and 53 ppm to 7.4 and 26 ppm, respectively.

## 4. Discussion

In this work, 34 strains belonging to *A*. section *Circumdati* were identified by conventional and molecular methods. According to morphological data, the tested population was characterised into four species namely: *A*. *insulicola*, *A*. *ochraceopetaliformis*, *A*. *ochraceus*, and *A*. *pseudoelegans*.

The morphological data of *A*. *insulicola* were partially in agreement with the description conducted by Visagie et al. (2014), who found that on MEA medium: the colony surface was floccose; mycelial areas white with sporulation converted into greyish orange; vesicle globose 15–30 μm wide, conidia globose to subglobose, smooth 2–3 × 2–3 μm. *A*. *ochraceopetaliformis* exhibited morphological characteristics similar with data obtained with Visagie et al. (2014), who reported that *A*. *ochraceopetaliformis* on MEA; colony surface floccose; white mycelial areas turned into olive brown with sporulation; reverse ranged from light brown to brown; reddish-brown sclerotium-like structures embedded in the medium; stipes ranged from hyaline to brown, rough walled 8–10 μm; vesicles pyriform to globose, 25–45 μm wide; conidia globose, smooth, 2–3 × 2–3 μm. Morphological characterisation of *A*. *ochraceus* was in full agreement with the result represented by Christensen (1982) and Visagie et al. (2014), they conducted that *A*. *ochraceus* on MEA; colony surface velutinous and floccose areas also appeared; dense sporulation varied from light to greyish yellow with white sterile mycelia; pinkish to purplish brown sclerotium; in almost strains vesicle typically globose; finely roughened conidia with globose to subglobose shape. The morphological criteria for *A*. *pseudoelegans* are in agreement with data obtained by Frisvad et al. (2004) and Visagie et al. (2014), they illustrated that *A*. *pseudoelegans* on MEA; colony surface floccose; mycelial area were white to light yellow; sparse conidiophores were formed after a long time of incubation; stipes rough, varied from hyaline to brown; vesicle globose to spathulate, 23–51 μm wide; conidia globose to subglobose, smooth, 2–3 × 2–3 μm; White to greyish sclerotia covered with mycelium were formed after a long time of incubation.

Molecular markers such as ITS, *β-tubulin*, and calmodulin gene were employed for *Aspergillus* species discrimination, but calmodulin gene was still the more informative gene and provided better resolution (Alshehri and Manikandan 2020; Susca et al. 2020). Based on calmodulin gene, the intelligible splitting of the tested strains into four different species. Various genetic regions were employed to discriminate *A*. section *Circumdati* into species levels. Using partial *β-tubulin* sequences, twenty different species were distinguished in this section and seven new species were recorded (Frisvad et al. 2004). Upon the ITS rDNA region, *BenA*, and partial *CaM* data, Visagie et al. (2014) concluded that *A*. section *Circumdati* contains 27 different species. According to the internal transcribed spacer (ITS), *A*. *curvatus* new species was recorded in *A*. section *Circumdati* (Al-Bedak et al. 2020).

In this work, we screened the detection of polyketide synthase gene (*pks*) in *A*. section *Circumdati* stains by using two-pair primers. 44.1% of tested strains were positive and exhibited one or two genes. All positive strains belonged to *A*. *ochraceus* and the rest of the fungal species failed to give any band. This finding was partially in agreement with data obtained by Dao et al. (2005) they reported that *A*. *melleus* NRRL 3519, *A*. *ochraceus* NRRL 3174, and *A*. *sulfureus* NRRL 4077 gave positive results with *AoLC*35-12 L/12 R primers. Also, El-Hamaky et al. (2016) found that exactly half of tested *A*. *ochraceus* strains were negative with *Aopks*1/2 primers. The genetic basis of OTA biosynthesis is far from being completely known (Gil-Serna et al. 2020), it consists of four genes encoding PKS, a non-ribosomal peptides synthetase (NRPS), a halogenase and a cytochrome P450 with transcription factor (bZIP) which consider specific for regulation of structural genes expression in *Aspergillus* species (Ferrara et al. 2016; Gil-Serna et al. 2018). Also in this situation, Alsalabi et al. (2023) confirmed the presence of *pks* gene in *A*. *ochraceus* by *AoOTAL*/*AoOTAR* and *Aopks*1/*Aopks*2 pairs and suggested that different genes were responsible for OTA synthesis.

Actually, 97% of *Aspergillus* population were ochratoxin producers (0.020 to 53 ppm) with the highest amount produced by IAEMAp1 strain belonging to *A*. *pseudoelegans*, followed by IAEMAi1 (*A. insulicola*) and IAEMAop2 (*A*. *ochraceopetaliformis*). *Aspergillus ochraceopetaliformis*, *A*. *ochraceus*, and *A*. *pseudoelegans* were previously reported as ochratoxin producers (Schmidt et al. 2003; Moslem et al. 2010; Visagie et al. 2014; Hareeri et al. 2022). To opposite with our data, Frisvad et al. (2004) and Visagie et al. (2014) reported that *A*. *insulicola* is not ochratoxins producer. Our data indicated different abilities of *A*. *ochraceopetaliformis* and *A*. *ochraceus* strains for OTA production, this finding agreed with Hashimoto et al. (2012), who screened 23 different strains of *A*. *ochraceus* for OTA production and found that 100% of tested strains were OTA producers with different levels. El-Hamaky et al. (2016) reported that 80% of tested *A*. *ochraceus* strains were capable of ochratoxin A production with amounts of 300–700 ppm. Several kinds of literature previously mentioned the different abilities of *A*. *ochraceus* group for OTA production (Bayman et al. 2002; Al-Said and El-Tedawy 2021).

Based on the results, ochratoxins levels in cultures of *A*. *insulicola* and *A*. *pseudoelegans* inoculated with ZnO-NPs were significantly decreased compared to control. The levels of Aflatoxins, ochratoxins, and fumonisins B1 were greatly depressed by ZnO-NPs applications (Hassan et al. 2013). The controlling of mycotoxins production by nanoparticles could be related to inhibition of fungal growth and modulation in gene expression (Deabes et al. 2018; Gómez et al. 2019).

In conclusion, four different species were discriminated in *A*. section *Circumdati* and *A*. *ochraceus* species constituted 79.4% of tested strains. Polypeptide synthase is not only the gene responsible for ochratoxins production in this section. ZnO-NPs have strong antimycotoxin activity.
